# [^18^ F] -FAPI-42 PET/CT assessment of Progressive right ventricle fibrosis under pressure overload

**DOI:** 10.1186/s12931-023-02565-5

**Published:** 2023-11-06

**Authors:** Xiaohui Zeng, Ruiyue Zhao, Zhixiong Wu, Zhuoji Ma, Chunxian Cen, Shanshan Gao, Wanxian Hong, Yanrong Yao, Kexin Wen, Shangwei Ding, Jian Wang, Wenju Lu, Xinlu Wang, Tao Wang

**Affiliations:** 1 State Key Laboratory of Respiratory Diseases, Guangdong Key Laboratory of Vascular Diseases, National Clinical Research Center for Respiratory Diseases, Guangzhou Institute of Respiratory Health, the First Affiliated Hospital of Guangzhou Medical University, Guangzhou, Guangdong China; 2 Department of Nuclear Medicine, the First Affiliated Hospital of Guangzhou Medical University, Guangzhou, Guangdong China; 3 Guangzhou Medical University, Guangzhou, Guangdong China; 4 Department of Ultrasound, the First Affiliated Hospital of Guangzhou Medical University, Guangzhou, Guangdong China

**Keywords:** Right Heart Failure, Fibrosis, RNA-sequence, Fibroblast activation protein, FAPI-PET/CT

## Abstract

**Background:**

Right heart failure (RHF) is a complication of pulmonary hypertension (PH) and increases the mortality independently of the underlying disease. However, the process of RHF development and progression is not fully understood. We aimed to develop effective approaches for early diagnosis and precise evaluation of RHF.

**Methods:**

Right ventricle (RV) pressure overload was performed via pulmonary artery banding (PAB) surgery in Sprague–Dawley (SD) rats to induce RHF. Echocardiography, right heart catheterization, histological staining, fibroblast activation protein (FAP) immunofluorescence and ^18^ F-labelled FAP inhibitor-42 ([^18^ F] -FAPI-42) positron emission tomography/computed tomography (PET/CT) were performed at day 3, week 1, 2, 4 and 8 after PAB. RNA sequencing was performed to explore molecular alterations between PAB and sham group at week 2 and week 4 after PAB respectively.

**Results:**

RV hemodynamic disorders were aggravated, and RV function was declined based on right heart catheterization and echocardiography at week 2, 4 and 8 after PAB. Progressive cardiac hypertrophy, fibrosis and capillary rarefaction could be observed in RV from 2 to 8 weeks after PAB. RNA sequencing indicated 80 upregulated genes and 43 downregulated genes in the RV at both week 2 and week 4 after PAB; Gene Ontology (GO) analysis revealed that fibrosis as the most significant biological process in the RV under pressure overload. Immunofluorescence indicated that FAP was upregulated in the RV from week 2 to week 8 after PAB; and [^18^ F] -FAPI-42 PET/CT revealed FAPI uptake was significantly higher in RV at week 2 and further increased at week 4 and 8 after PAB.

**Conclusion:**

RV function is progressively declined with fibrosis as the most prominent molecular change after pressure overload, and [^18^ F] -FAPI-42 PET/CT is as sensitive and accurate as histopathology in RV fibrosis evaluation.

**Supplementary Information:**

The online version contains supplementary material available at 10.1186/s12931-023-02565-5.

## Background

Pulmonary hypertension (PH) is a progressive disease with poor prognosis, which affects approximately 1% people worldwide according to recent epidemiological data [1, 2]. In PH, the increased pulmonary vascular resistance results in chronic pressure overload to right ventricular (RV) and leads to right heart failure (RHF). The survival rate for pulmonary arterial hypertension (PAH) patients with stable RV function is much higher than patients with devastating RV function, independent of pulmonary vascular resistance, and RHF is the major cause of mortality and morbidity among PH patients [3]. Because of the irregular size of RV, it is technically difficult to evaluate RHF precisely.

In the presence of pressure overload, RV cardiac fibroblasts activate and subsequently remodel the myocardium via pathological alterations of collagen network that surrounds cardiomyocytes and interstitium, which leads to myocardial fibrosis and reduced compliance of the RV [4]. Currently, myocardial fibrosis is assessed by magnetic resonance imaging (MRI) and endomyocardial biopsy [5]. However, cardiac fibrosis cannot be detected by MRI at the early stage of heart failure [6], and endomyocardial biopsy is invasive. Fibroblast activation protein (FAP) is robustly expressed in activated fibroblast, and could be detected by FAP inhibitor-42 (FAPI-42) via positron emission tomography (PET)/computed tomography (CT) imaging [7]. Currently, FAPI PET/CT has been used to identify fibroblast activation in the process of lung and liver fibrosis [8, 9], and a recent image study reported higher uptake of FAPI in the RV of a PH patient with PET/CT imaging [10, 11]. However, the effects of [^18^ F] ‑FAPI-42 PET/CT for evaluating the progress of RHF, especially the early stage of RHF, have not been reported.

In addition to myocardial fibrosis, the other pathological changes of RV failure include hypertrophy, inflammation and capillary rarefaction; while molecular changes include alteration in the expression of genes related to extracellular matrix, inflammation, angiogenesis, mitochondrial biogenesis and reactive oxygen species production [12]. Despite of these understandings, there is a lack of approaches for early diagnosis of RHF, and there is no medical treatment that directly targets RV failure so far [13]. In an attempt to explore novel mechanisms that may lead to early diagnosis and precise evaluation of RHF, this study is aimed to demonstrate the process of RV failure development and progression in response to pressure overload. Here, using surgical pulmonary artery banding (PAB) to establish an RV pressure overload rat model, we sought to demonstrate the longitudinal echocardiographic, hemodynamic, histological, and molecular changes of RVF, and determine the dynamic changes of [^18^ F] -FAPI-42 uptake via PET/CT at a series of time points.

## Methods

### Animals and pulmonary artery banding models

**Fig. 1:**
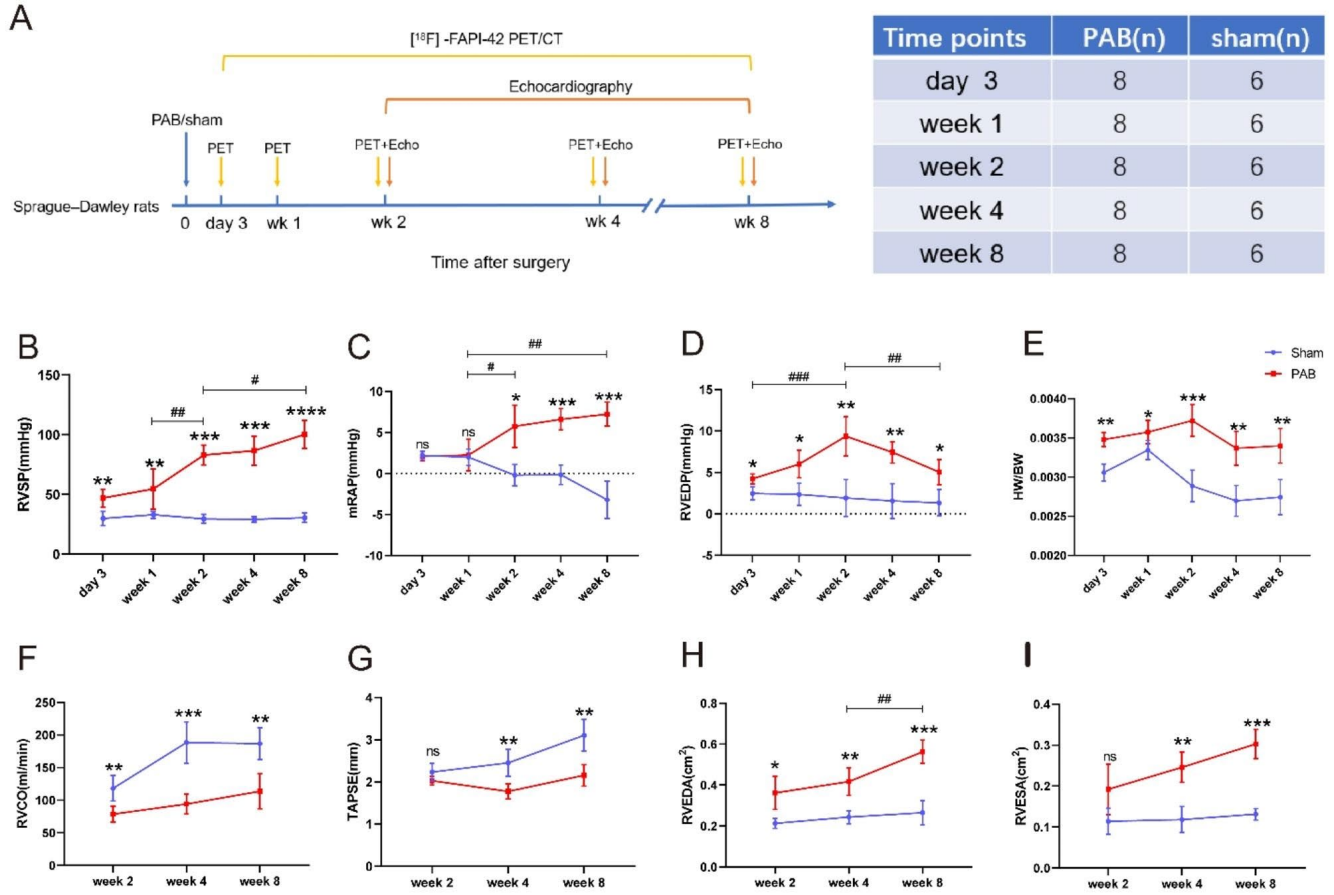
Structural and functional disorders of the right ventricle under pressure overload. (**A**) Study schema for PAB model. (**B**)-(**D**) Right heart catheterization suggests increased RVSP, mRAP and RVEDP. (**E**) Ratio of the heart weight to body weight. (**F**-**I**) Echocardiography indicates enlarged right ventricular (RV) cavity and impaired RV contractility in PAB rats (n = 5–7/group). ns = not significant, *p < 0.05, **p < 0.01, ***p < 0.001, ****p < 0.0001, compared with sham group. # *p* < 0.05, ## *p* < 0.01, ### *p* < 0.001, compared with the indicated time point in PAB group. RVSP = right ventricular systolic pressure, mRAP = mean right atrial pressure, RVEDP = right ventricular end-diastolic pressure, HW/BW = heart weight/ body weight, RVCO = right ventricular cardiac output, TAPSE = tricuspid annular plane systolic excursion, RVEDA = right ventricular end-diastolic area, RVESA = right ventricular end-systolic area, PAB = pulmonary artery banding group, sham = sham group

Male Sprague–Dawley (SD) rats (190–220 g) were purchased from Guangdong Provincial Medical Experimental Animal Center (Guangzhou, China). Rats were randomly divided into pulmonary artery banding (PAB) group (n = 8 per time point, a total of 40 rats were included for the 5 time points) and sham group (n = 6 per time point, a total of 30 rats were included for the 5 time points). PAB was performed according to the following protocol. After being anesthetized by isoflurane, rats were intubated and attached to a ventilator (HX-101E, Techman, China). Then, a left thoracotomy was performed to expose the pulmonary artery trunk. To construct a constant narrowing, the pulmonary artery trunk was banded with a 4–0 suture by tying over an 18-gauge needle. The sham rats underwent the same surgical procedures without pulmonary artery banding. Post-operative pain relief was provided with ibuprofen supplemented water for 7 consecutive days. All animals were housed in a specific pathogen-free room with free access to water and food. All experiments were executed in accordance with the rules and guidelines approved by the Animal Care and Use Committee of Guangzhou Medical University. The timeline and number of the animals were demonstrated in Fig. 1A.

### Hemodynamic assessment

Hemodynamic measurements were performed at day 3, week 1, 2, 4 and 8 after PAB or sham surgery, as previously described [14]. Briefly, right ventricular systolic pressure (RVSP), mean right atrial pressure (mRAP) and right ventricular end-diastolic pressure (RVEDP) were measured using a 1.4-F micro-tip pressure transducer catheter (Millar Instruments, Houston, TX, USA) connected to a PowerLab data acquisition system (AD Instruments, Sydney, Australia).

### Echocardiography

Echocardiography was performed 2, 4 and 8 weeks after surgery using Philips EPIQ 7 C (Philips Healthcare, Shanghai, China) Doppler ultrasound machine equipped with an S8-3 transducer (3–8 MHz). Rats were anaesthetized using 1.5% isoflurane and then fixed in supine position before echocardiography assessment. The measurement method of RV function parameters refers to the 2015 ASE Adult Echocardiography Cardiac Chamber Quantification Guidelines. Briefly, we displayed the apical four-chamber view of the RV and measured the RV function parameters, including right ventricular cardiac output (RVCO), tricuspid annular plane systolic excursion (TAPSE), right ventricular end-diastolic area (RVEDA), right ventricular end-systolic area (RVESA); and displayed the long axis view of pulmonary artery to measure the pulmonary artery diameter (PAd) and achieve the flow spectrum at the site of pulmonary artery stenosis (FSpas), and measured its velocity time integral (VTI), calculated RVCO = VTI × π × PAd²×HR/4.

### Histology and immunofluorescence

To assess RV histological changes under pressure overload, ventricle tissue was collected from PAB or sham rats at day 3, week 1, week 2, week 4, week 8 post-surgery, respectively. After collection, the heart from each animal were flushed with saline, and then fixed in 10% formaldehyde for 24 h. After paraffin embedding, sections were cut at 5 μm thickness for hematoxylin and eosin (HE) or Masson’s trichrome staining. Cardiomyocyte hypertrophy was assessed by measuring the cross-sectional area of cardiomyocytes, twenty cardiomyocytes were randomly selected from each RV to calculate the mean cross-sectional area of cardiomyocytes. And RV fibrosis was quantitated via calculating the ratios of the fibrosis area to the total area of RV free wall based on Masson’s trichrome staining. The immunofluorescence (IF) staining was performed as previously described [15]. Briefly, after deparaffinization and antigen retrieval, heart sections were blocked with 5% BSA for 1 h at room temperature and then incubated with primary antibody at 4 °C overnight, followed with species-specific Alexa Fluor-coupled secondary antibodies at room temperature for 2 h. The information of primary antibodies is displayed as follows: anti-CD31 (1: 500, GB12063, Servicebio, China), anti-FAP (1: 100, A6349, ABclonal, China). Image analysis was performed using Image J software (NIH, Bethesda, MD, USA).

### Quantitative RT-PCR (qPCR)

Total RNA was isolated from RV tissue of PAB or sham rats using Trizol Reagent (Invitrogen, USA) and then reverse transcribed into cDNA using Prime Script RT Kit (Takara). Next, qPCR assay was performed on a Bio-rad CFX CONNECT detector system using SYBR Green universal PCR mix (Takara). The 18 S ribosomal RNA was used as internal reference. The relative expression analysis was calculated with reference to the 2^−ΔΔCt^ method as previously described [16, 17]. The primers used in the test were purchased from Sangon Biotech (Shanghai, China) and the sequences were listed in Supplemental Table 1.

### RNA sequencing (RNA seq) and bioinformatics analysis

The RNA seq procedures were performed as previously described [18]. Briefly, ribosome RNA Depletion Kit v2 (E7400L, NEBNext, MA, USA) was used to remove the ribosomal RNA (rRNA) from total RNA. Next, RNA seq libraries preparation was generated using Ultra II RNA Library Prep Kit (E7770s, NEBNext, MA, USA) and quantified using the Agilent 4200 Bioanalyzer (Shanghai, China). RNA-sequencing was executed in the PCR Lab, State Key Laboratory of Respiratory Disease, Guangzhou Medical University. Concisely, the RNA-seq libraries were prepared according to the standard Illumina protocol. Paired-end sequencing at 150 bp length and depth of 34X was performed on NextSeq 550AR (Illumina Inc, CA, USA). Data quality checks were performed on the Analysis Viewer and demultiplexing was performed with Bcl2fastq (Illumina Inc, CA, USA). Paired-end RNA sequencing reads were analyzed using open-source software tools fastp, HISAT2, SAM tools, and feature Counts. The DESeq2 package was used to identify deferentially expressed genes (DEGs) with an adjusted P value less than or equal to 0.05. Genes with a fold change (FC) above 1.2 were considered up-regulated and genes with a FC below 0.83333 were considered down-regulated. DEGs were subjected to Gene Ontology (GO) enrichment analyses as described in previous study [19]. Log_2_FC of all genes were shrunk using ashr (Adaptive Shrinkage, using Empirical Bayes) package, and all genes ranked with shrunk log_2_FC were used for gene sets enrichment analysis (GSEA) using cluster Profiler package on custom-defined gene sets curated from KEGG.

### [^18^ F] ‑FAPI-42 PET/CT imaging and quantitative analysis

Synthesis of [^18^ F] -FAPI-42 was performed as previously reported [20]. All the imaging examinations were performed on PET/CT (MadicLab PSA071, Shandong Madic Technology Co.,Ltd., China), the PET/CT image was collected 40–50 min after injection with 7.4 ± 1.8 MBq of [^18^ F] -FAPI-42. The entire PET/CT scan was performed while the rat was under 1.5% isoflurane anesthesia. PET/CT images were reconstructed in 3D RAMLA with CT scan (80 kV, 70 mAs) for attenuation correction and fusion localization, with 0.8 mm × 0.8 mm × 0.8 mm as the final resolution. All data was processed and images were analyzed with software PMOD (version 4.3, PMOD Technologies Ltd., Zurich, Switzerland). Volume of interests (VOI) in the heart, pulmonary orifice and muscle in the left foreleg were drawn manually according to the PET/CT imaging. To eliminate interference imaging and make sure the images were from the right heart, cross section and coronal section of imaging were performed. The standardized uptake value (SUVmax) of [^18^ F] -FAPI-42 in the VOIs were calculated, and the ratios of SUV (SUVR) were calculated by the ratio of target uptake to reference uptake.

### Statistical analysis

The data were expressed as mean ± standard deviation. Statistical analysis was performed using GraphPad Prism 8.0 software. An unpaired t-test was used between sham and PAB groups, and comparisons over time within PAB group were tested with one-way ANOVA followed by Bonferroni post-hoc test or Kruskal-Wallis test as appropriate. Correlation was evaluated by Spearman’s method. *p* values < 0.05 was considered statistically significant.

## Results

### Progressive RV hypertrophy and dysfunction after pressure overload

Compared to sham operated, there was a significant increase of RVSP and mRAP since week 1 after PAB, and the upward trend was expanding with the duration of PAB until the end of our experiment (week 8 after PAB) (Fig. 1B and C); and RVEDP began to increase at day 3 and peaked at week 2 after PAB (Fig. 1D). The heart-to-body weight ratio was increased in PAB compared to sham-operated rats at day 3, week 1, week 2, week 4 and week 8 after surgery (Fig. 1E). Echocardiography at week 2, 4 and 8 showed decreased RVCO and TAPSE (Fig. 1F and G), and increased RVEDA and RVESA in PAB rats (Fig. 1H and I). These data demonstrate that RV pressure and size were increased, and RV function was reduced gradually with the duration of pressure overload.

### Pressure overload leads to Progressive myocardial hypertrophy, fibrosis and capillary rarefaction in RV

**Fig. 2:**
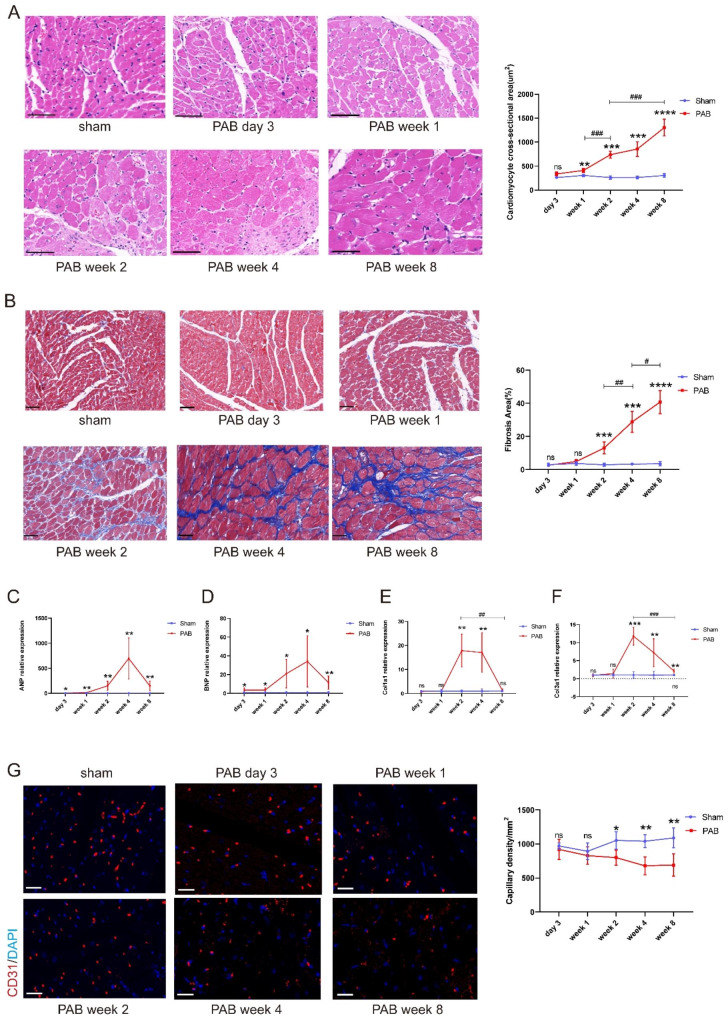
Progressive myocardial hypertrophy, fibrosis and capillary rarefaction were observed in the RV from PAB rats. (**A**) Hematoxylin-eosin (HE) staining reveals larger cross-sectional area of cardiomyocytes in the RV from PAB rats (left); quantification of RV cross-sectional area of cardiomyocytes in PAB and sham rats at different time points (right). (**B**) The PAB rats demonstrate significant RV fibrosis starting from 2 weeks after surgery by Masson’s trichrome staining (left); quantification of RV fibrosis area in rats (right). (**C**)-(**D**) Compared with sham group, ANP and BNP expression in RV tissue of PAB rats were elevated and reach the peak at 4 weeks post-PAB by qPCR. (**E**)-(**F**) Fibrosis markers (Col1a1 and Col3a1) were increased in the RV of PAB rats and reach the peak at week 2 after surgery by qPCR. (**G**) Representative figures of CD31 immunofluorescence in RV (left), CD31(red) = platelet endothelial cell adhesion molecule; DAPI (blue) = 4′,6-diamidino-2-phenylindole. Quantitative analysis of RV capillary density (right). RV capillaries in PAB were decreased gradually from week 2 to 8 after surgery. (n = 5–7/group). ns = not significant, *p < 0.05, **p < 0.01, ***p < 0.001, ****p < 0.0001, compared with sham group. # *p* < 0.05, ## *p* < 0.01, ### *p* < 0.001, compared with the indicated time point in PAB group. bar = 50 μm for HE and Masson’s trichrome staining, bar = 20 μm for CD31 immunofluorescence staining. ANP = atrial natriuretic peptide, BNP = brain natriuretic peptide, Col1a1 = collagen 1a1, Col3a1 = collagen 3a1, PAB = pulmonary artery banding group, sham = sham group

Myocardial hypertrophy of the RV was assessed by mean cross-sectional area of cardiomyocytes based on HE staining, and there was no significant change in the RV at day 3 after PAB, but RV cardiomyocytes hypertrophy gradually increased with the duration of PAB compared to the sham group (408.7 ± 41.22 µm^2^ vs. 303.6 ± 24.6µm^2^, *p* < 0.01, at week 1; 738 ± 73.91 µm^2^ vs. 260 ± 36.06 µm^2^, *p* < 0.001, at week 2; 855 ± 151.9 µm^2^ vs. 263.2 ± 27.73 µm^2^, *p* < 0.001, at week 4; 1302.2 ± 174.7 µm^2^ vs. 302.2 ± 38.07 µm^2^, *p* < 0.0001, at week 8) (Fig. 2A). Formation of RV fibrotic lesion was assessed by collagen deposition within the myocardium via Masson’s trichrome staining, which showed significant RV fibrosis since week 2 and progressed over time in PAB rats (Fig. 2B). In addition, we also assessed cardiac hypertrophy and fibrosis in the free wall of left ventricle, and found no significant alterations of these indices at any time point after PAB (Supplemental Fig. 2). The molecular indicators of heart failure (ANP and BNP) and fibrosis (Col1a1 and Col3a1) were elevated in the RV of PAB rats compared to sham rats. ANP and BNP were increased at day 3 and peaked at week 4 after PAB (Fig. 2 C and D); while Col1a1 and Col3a1 elevation reached the peak at week 2 and gradually decreased at week 4 and 8 after PAB (Fig. 2E and F). Previous study has revealed that endothelial-mesenchymal transition (EndMT) contributes to the deposition of collagen in the heart [21]. To investigate whether fibrosis in PAB RV is associated with EndMT, we examined EndMT markers by qPCR. The ratio analysis of mesenchymal cell markers (Fibronectin and Vimentin) to endothelial cell markers (CD31 and VE-cadherin) shows that Vimentin/CD31 and Vimentin/VE-cadherin were increased since week 2 after PAB when compared to the sham group at each time point, respectively. These may suggest that the deposition of collagen in RV is partially from EndMT. (Supplemental Fig. 3). As capillary rarefaction in RV leads to decompensated heart failure, we performed CD31 immunofluorescence staining to detect capillaries. Compared to the sham group, RV capillary density in PAB rats was not significantly changed in the early stage of PAB (3 days and 1week after surgery). However, reduction in RV capillary density were found at week 2 (*p* = 0.013) and became more prominent at week 4 (*p* = 0.0012) and week 8 (*p* = 0.0037) after PAB (Fig. 2G).

### Myocardial fibrosis is the most prominent molecular change in RHF

**Fig. 3:**
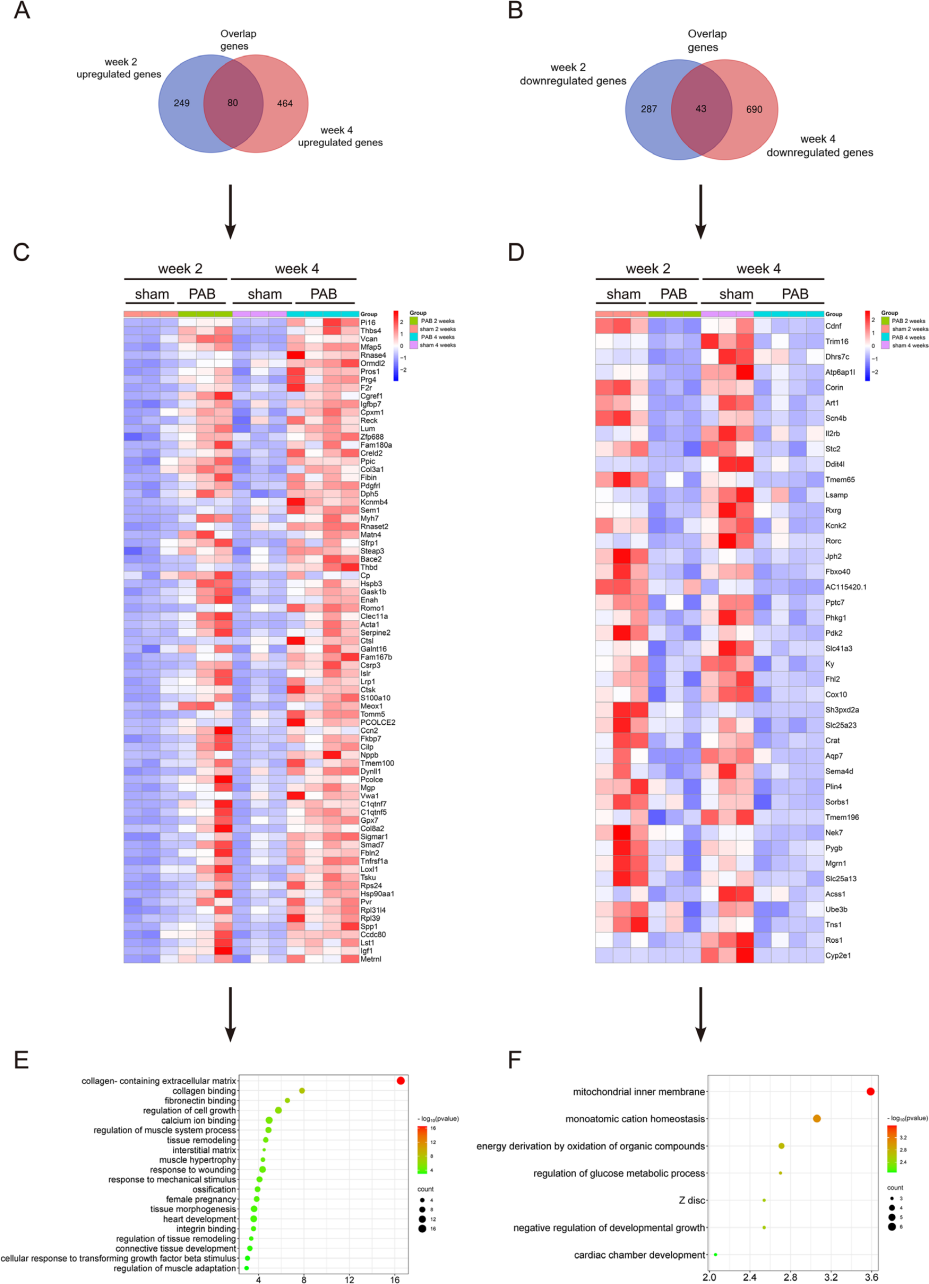
RNA sequencing indicates that myocardial fibrosis is a critical process in RV lesions in PAB rats. (**A**)-(**B**) Venn diagram demonstrating differentially expressed genes (DEGs) in the RV tissue between PAB and sham rats at week 2 and week 4, indicating 329 and 544 up-regulated genes at week 2 and 4 respectively (A), 330 and 733 down-regulated genes at week 2 and 4 respectively (**B**) (FC cut-offs > 1.2 or < 0.83333). (**C**)-(**D**) Heatmap illustrating the overlapped DEGs from week 2 and week 4, indicating 80 up-regulated genes (**C**) and 43 down-regulated genes (**D**) at both time points. (**E**) The top 20 GO (Gene Ontology) items of the overlapped upregulated genes are displayed and the GO items of extracellular matrix and fibrosis are most significantly enriched in the RV from PAB rats. (F) The GO analysis on the overlapped downregulated genes. PAB = pulmonary artery banding group, sham = sham group

In order to identify the molecular changes in the process of RHF, we performed RNA-sequencing on RV tissue samples at week 2 and week 4 after PAB. Compared to the sham group, there were 329 genes and 544 genes upregulated in PAB group at week 2 and week 4, respectively; and 330 genes and 733 genes downregulated in PAB group at week 2 and week 4, respectively. (Figure. 3 A and B, Fold Change cut-offs > 1.2 or < 0.83333). With an attempt to study the genes that are essential for the intrinsic process of RHF development and progression, we analyzed the overlapped genes from the 2 time points, and identified 80 upregulated genes and 43 downregulated genes that were both regulated at the same direction at week 2 and 4 after PAB (Fig. 3 C and D). Gene Ontology (GO) analysis on the overlapped upregulated genes showed “collagen-containing extracellular matrix”, “collagen binding” and “fibronectin binding” as the top 3 biological processes (Fig. 3E). In addition, Gene Set Enrichment Analysis (GSEA) showed that “extracellular matrix” was significantly enriched in the PAB group (ES = 0.521, *p* = 0.0446) (Supplemental Fig. 1A). As fibrosis is characterized by excessive accumulation of collagen and other extracellular matrix components, the above data indicated that myocardial fibrosis plays a vital role in the process of pressure overload-induced RHF. GO analysis on the overlapped downregulated genes showed “mitochondrial inner membrane”, “monoatomic cation homeostasis” and “energy derivation by oxidation of organic compounds” as the top 3 GO items (Fig. 3F), which is consistent with the previous reports that pressure overload-induced RV failure is associated with decreased energy generation [22].

### FAP is progressively increased in RV with the duration pressure overload

**Fig. 4:**
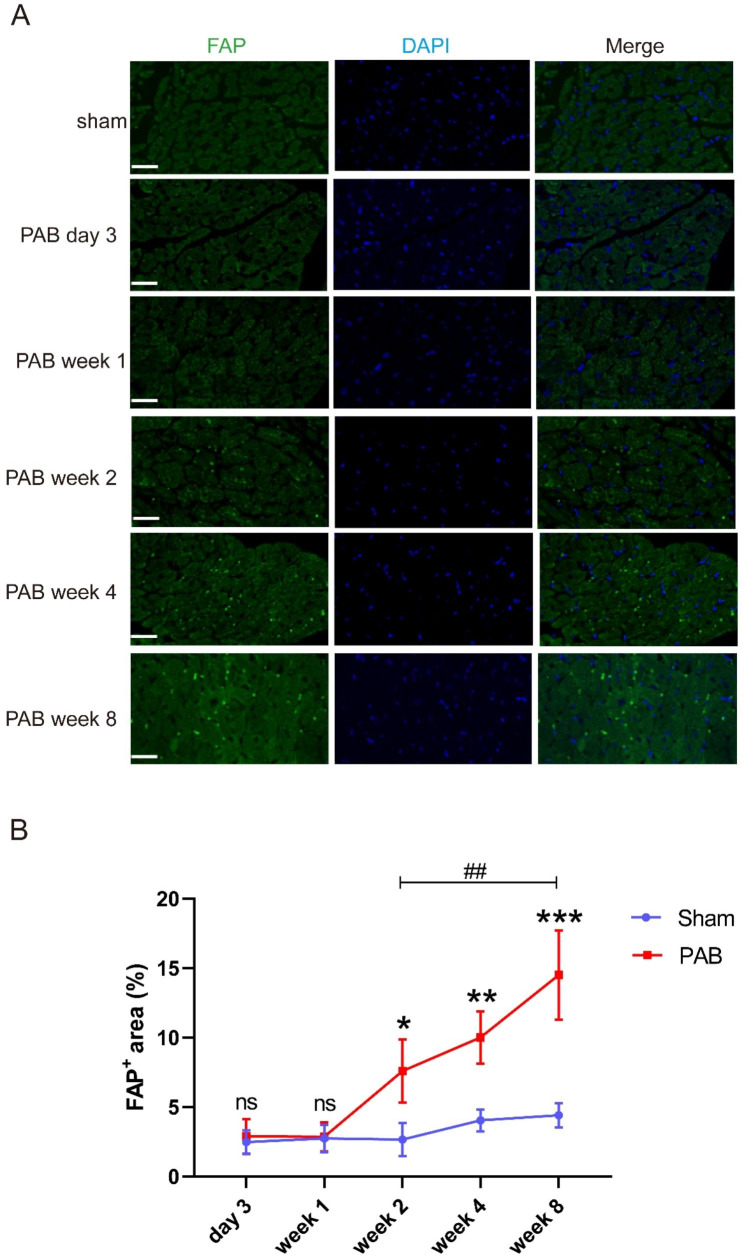
Immunofluorescence staining reveals that FAP is progressively increased in RV with the duration pressure overload. (**A**) Representative FAP immunofluorescence pictures of RV from sham or PAB rats (day 3, week 1, week 2, week 4 and week 8). FAP (green) = Fibroblast Activation Protein; DAPI (blue) = 4′,6 -diamidino-2-phenylindole. (**B**) Quantitative analysis of FAP-positive area in RV: no significant increase of FAP-positive cells in early PAB (day 3 and week 1 after surgery), FAP began to be detected at week 2 and progressively increased until the end of the experiment (week 8). ns = not significant, *p < 0.05, **p < 0.01, ***p < 0.001, compared with sham group. ##*p* < 0.01, compared with the indicated time point among PAB group. n= (5–7/group), bar = 25 μm, PAB = pulmonary artery banding group, sham = sham group

Having found that myocardial fibrosis is the key process of RHF, we next sought to explore approaches to evaluate RV fibrosis. FAP is increased in the process of fibrogenesis, and radiolabeled FAPI has been developed to detect tissue FAP to evaluate organ fibrosis [23, 24]. Among our differential expression genes (DEGs), FAP expression was increased significantly in the RV of PAB rats at week 4 when compared to the sham operated (Supplemental Fig. 1B). Immunofluorescence of RV tissue indicated that FAP was upregulated at week 2 and 4, and the upregulation was further increased at week 8 after PAB when compared to sham operated rats (Fig. 4A and B).

**Fig. 5:**
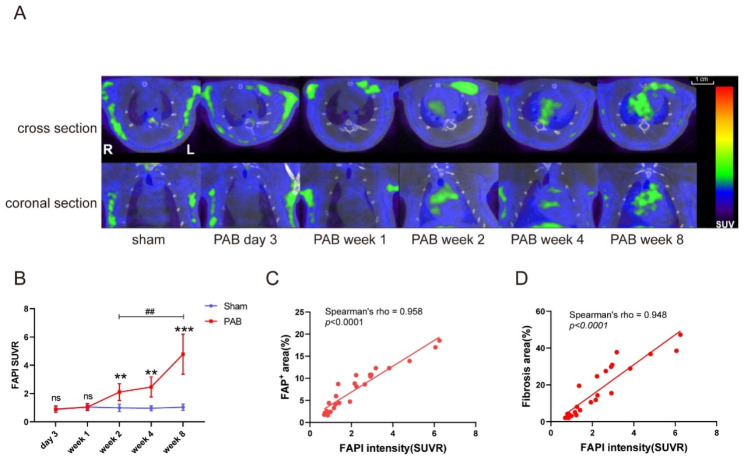
FAPI signal in RV is progressively increased from PAB rats by [^18^ F] -FAPI-42 PET/CT imaging. (**A**) Representative [^18^ F] -FAPI-42 PET/CT images in right heart. Similar with the sham rats, no significant [^18^ F] -FAPI-42 uptake was observed at day 3 or week 1 after PAB. Pressure overload resulted in increased FAPI signal from week 2 to week 8 after PAB by [^18^ F] -FAPI-42 PET/CT. (**B**) SUVR of [^18^ F] -FAPI-42 in the right heart from sham and PAB rats. (**C**)-(**D**) The intensity of [^18^ F] -FAPI PET in right heart directly correlated with the FAP immunofluorescence (**C**) and the RV fibrosis detected by Masson’s trichrome staining (**D**). n= (5/group), ns = not significant, **p < 0.01, ***p < 0.001, compared with sham group. ##*p* < 0.01, compared with the indicated time point of PAB group. SUVR = ratios of standardized uptake value, R = right, L = left, PAB = pulmonary artery banding group, sham = sham group

To monitor the progression of RV fibrosis in vivo, we performed [^18^ F] -FAPI-42- PET/CT at a series of time points after PAB. At day 3 and week 1 after PAB, no obvious [^18^ F] -FAPI uptake was observed in the right heart; but [^18^ F] -FAPI uptake was higher in RV at week 2 after PAB when compared to sham-operated (SUVR = 2.104 ± 0.598 vs. 0.99 ± 0.251, *p* = 0.005), and further increased at week 4 (SUVR = 2.466 ± 0.7106 vs. 0.961 ± 0.1763, *p* = 0.0018) and week 8 (SUVR = 4.782 ± 1.416 vs. 1.035 ± 0.2162, *p* = 0.0004) with the duration of pressure overload (Fig. 5A and B). Moreover, the intensity of [^18^ F]-FAPI PET in right heart directly correlated with the FAP immunofluorescence (Spearman’s rho = 0.958, *p* < 0.0001) and Masson’s trichrome staining (Spearman’s rho = 0.948, *p* < 0.0001) of RV tissue (Fig. 5 C and D). Together, these may suggest that [^18^ F] -FAPI-42-PET/CT is as sensitive and accurate as tissue histology in RV fibrosis detection, and could be used as a noninvasive imaging tool for early diagnosis and precise evaluation of RHF.

## Discussion

This study demonstrated that RV myocardial hypertrophy, RV fibrosis and capillary rarefaction emerged early after PAB and gradually progressed with the duration of pressure overload, which suggest that pressure overload-induced RV dysfunction is progressive. Using RNA-seq as an unbiased tool, we identified that genes related to fibrosis is the most critical process of RHF. Inspired by these findings, we investigated the changes of FAP which has been developed as an imaging maker of fibrosis, and found that the uptake of [^18^ F]-FAPI-42 was gradually increased via PET/CT imaging with the duration of pressure overload; in addition, the uptake of [^18^ F]-FAPI-42 is RV is associated with cardiac fibrosis detected by tissue staining. To best of our knowledge, this is the first study that applied [^18^ F]-FAPI-42-PET/CT as a noninvasive tool for precise evaluation of RHF development and progression.

In response to pressure overload, the changes of RV structure include cardiomyocyte hypertrophy, fibrosis and capillary rarefaction, which termed as RV remodeling. In our study, we found that RV hypertrophy appears earlier than fibrotic and vascular myocardial remodeling in rat PAB model, which is consistent with the results from other RV pressure overload models which are mainly PH animal models [25]. Compared with the RV pressure load study in PH animal models induced by monocrotaline, hypoxia or SU5416 + hypoxia, the RV remodeling in PAB models is purely dependent of pressure overload, as drugs or hypoxia may lead to direct damage on RV function [26]. Interestingly, we found that the RVEDP began to increase at day 3 and peaked at week 2 after PAB, then gradually decreased at 4 and 8 weeks. This result is consistent with a previous report which shows that RVEDP peaked at week 2, and gradually decreased at week 5 and 12 post-PAB in rats [27]. The following reasons may contribute to the decline in RVEDP after 2 weeks: first, the rats used in our study were 6-week-old, which is still growing at the beginning of the experiment, and the growth of body and heart at the later time points may affect RVEDP; second, the degree of anesthesia could be lower in the later time points in our experiment, which may reduce RVEDP. These speculations were supported by the fact that the rats in the sham group also showed lower RVEDP at week 4 and 8 than those at week 2.

Using an unbiased RNA sequencing analysis, we found that the most enriched GO terms of the key upregulated genes in the RV from PAB models are associated to collagen fiber deposition, which is an important process of tissue fibrosis. RV fibrosis is important for PH prognosis, PAH patients with systemic sclerosis which is characterized by excessive fibrosis in the body including RV have a shorter survival duration [28]; in the contrary, PAH patients with Eisenmenger syndrome show less RV fibrosis and have a longer survival duration, when compared to idiopathic PAH [29]. These may suggest that excessive myocardial fibrosis in RV implicates worse prognosis in RHF. Pathophysiologically, myocardial fibrosis leads to RV stiffness [25], and diminished diastolic and systolic function of RV, which may eventually result in decompensated RHF. Cardiomyocytes have been found as the cell type that immediately respond to pressure overload in RV, which has been considered as compensative because hypertrophic RV could overcome the increased pulmonary vascular resistance to maintain sufficient cardiac output. As the pressure overload persists, RV hypertrophy transits to RHF, and RV fibrosis is a remarkable feature in decompensated RHF [30]. In a PAB model with gradual reduction of the afterload burden through absorption of pulmonary artery bands, normalization of cardiomyocyte hypertrophy was observed at earlier time point compared to the reversal of myocardial fibrosis [31]. These may suggest that if myocardial fibrosis appears in RV, it may take longer duration for PH patients to recover with afterload reduction. Thus, it is important to determine RV fibrosis in PH patients, which may help the PH specialists to evaluate the prognosis of the disease.

Currently, there is no ideal technique for assessing RV fibrosis effectively in RHF patients. Myocardial fibrosis is mainly assessed by magnetic resonance imaging (MRI) and endomyocardial biopsy [5]. Nevertheless, myocardial fibrosis cannot be detected by MRI at the early stage of heart failure, and endomyocardial biopsy is invasive. Several case reports revealed that the right heart uptake of ^68^Ga-FAPI was observed in patients with idiopathic PAH and chronic thromboembolic pulmonary hypertension (CTEPH) [11, 32]. However, it remains unknown whether FAPI-PET/CT can be used to estimate the severity of RV fibrosis or detect RV fibrosis as early as tissue biopsy. Compared to ^68^Ga (t_1/2_=67.8 min), which is typically produced in limited quantities and has a short half-life, ^18^ F (t_1/2_=109.7 min) offers greater convenience in terms of transportation and availability from cyclotrons, and its lower positron energy (^18^ F 0.25 MeV vs. ^68^Ga 0.83 MeV) provides higher spatial resolution in PET/CT imaging. The ^18^ F-labeled FAPI targeted radioligand, ^18^ F-FAPI-42, has been reported following to exhibit comparable uptake in FAP-positive cells and tumors as ^68^Ga-FAPI-04 [33], with numerous studies demonstrating its diagnostic efficacy in various cancers and fibrotic diseases [34–36]. In our study, using a noninvasive [^18^ F] -FAPI-42-PET/CT imaging, we could identify obvious RV fibrosis 2 weeks after PAB, and this time point is consistent with our pathological finding of myocardial fibrosis from RV specimen. Moreover, correlation analysis shows that RV uptake of [^18^ F] -FAPI-42 in PET/CT is positively associated with collagen deposition detected by Masson’s Trichrome staining of RV specimen with the progression of RHF. These may suggest that [^18^ F] -FAPI-42-PET/CT is as accurate as tissue biopsy in evaluating RV fibrosis induced by pressure overload, even at the early phase of RHF. Pulmonary arterial adventitial fibroblast (PAAF) plays an essential role in pulmonary artery remodeling during the progression of PH. PAAF activation deteriorates PH through reduction of pulmonary artery compliance and induction of perivascular inflammation [37, 38]. Using ^68^ Ga-FAPI-04 PET/CT in thirteen patients with CTEPH, nine (69%) patients showed enhanced ^68^ Ga-FAPI-04 uptake in the main pulmonary artery (PA) and PA branches, and ^68^ Ga-FAPI-04 activity in PA is positively correlated with pulmonary diastolic pressure [39]. Together with our findings in RV, these suggest that FAPI-PET/CT has the potential to assess fibroblast activation in both RV and PA, as RV-PA coupling is vital to the prognosis of PH patients [40], measurement of fibrotic remodeling in both RV and PA may provide a new perspective for the evaluation of PH.

Current RHF therapies rely on load amelioration, and there is no approved therapy that directly improve RV function. As fibrosis is the most prominent molecular changes and positively correlated with the progression of RHF; thus, inhibition of myocardial fibrosis remodeling is a potential strategy to preserve RV function. In this study, we found that FAP is significantly increased after PAB and correlates with myocardial fibrosis detected by RV pathological staining. Indeed, FAP is not only a marker of tissue fibrosis, but also participates in fibrogenesis. In mice myocardial infarction model, inhibition of FAP reduces ventricular fibrosis, improves cardiac function and promotes angiogenesis via stabilizing BNP [41]. And pharmacological FAP inhibition alleviates liver fibrosis in chronic liver injury via attenuating macrophage infiltration and activation [42], macrophage-derived inflammation contributes to the development of RHF [43]. The above may suggest that targeting FAP may change the prognosis of RHF, which is worth to be investigated in the future. In addition, GO analysis shows that transforming growth factor-β (TGF-β) cascade was enriched among the upregulated genes in the RV tissue from PAB rats (Fig. 3E). As activation of TGF-β pathway has been reported to exacerbate left ventricular fibrosis under pressure overload [44], these may suggest that TGF-β pathway contributes to RHF development. Recent clinical trials indicated that sotatercept, a drug that targets TGF-β pathway, reduces pulmonary vascular resistance among PH patients [45]. Thus, this drug may benefit PH patients in both pulmonary vascular resistance amelioration and RV function preservation via inhibiting TGF-β pathway.

Several limitations should be considered in this study. First, only male rats were used to induced PAB models; we choose the male in this study because female rats show better adaptation to RV afterload burden [46]. Second, we only observed the RV changes in 8 weeks after PAB because there is a high mortality rate after 8 weeks due to severe RHF. Third, the results in this study should be considered as a proof-of-principle study, more mechanistic studies are warranted to demonstrate the mechanism of fibrosis and FAP changes in RV under pressure overload.

## Conclusions

Our study found that RV function is progressively declined with fibrosis as the most prominent molecular changes with the duration of pressure overload, [^18^ F] -FAPI-42 PET/CT is as sensitive and accurate as histopathology in RV fibrosis evaluation.

### Electronic supplementary material

Below is the link to the electronic supplementary material.

Supplementary Material 1

Supplementary Material 2

Supplementary Material 3

Supplementary Material 4

## Data Availability

The data and materials that support the findings of this research are available from the corresponding author upon reasonable request.

## Abbreviations

RHF: Right heart failure
PH: Pulmonary hypertension
RV: Right ventricle
PAB: Pulmonary artery banding
FAP: Fibroblast activation protein
PET/CT: Positron emission tomography/computed tomography
RVSP: Right ventricular systolic pressure
mRAP: Mean right atrial pressure
RVEDP: Right ventricular end-diastolic pressure
RVCO: Right ventricular cardiac output
TAPSE: Tricuspid annular plane systolic excursion
RVEDA: Right ventricular end-diastolic area
RVESA: Right ventricular end-systolic area
SUVR: Ratios of standardized uptake value
