# Supplementary figures and images for: [18 F] -FAPI-42 PET/CT assessment of Progressive right ventricle fibrosis under pressure overload
Source: Respir Res. 2023 Nov 6;24:270. doi: 10.1186/s12931-023-02565-5 (PMC10626814; doi:10.1186/s12931-023-02565-5)

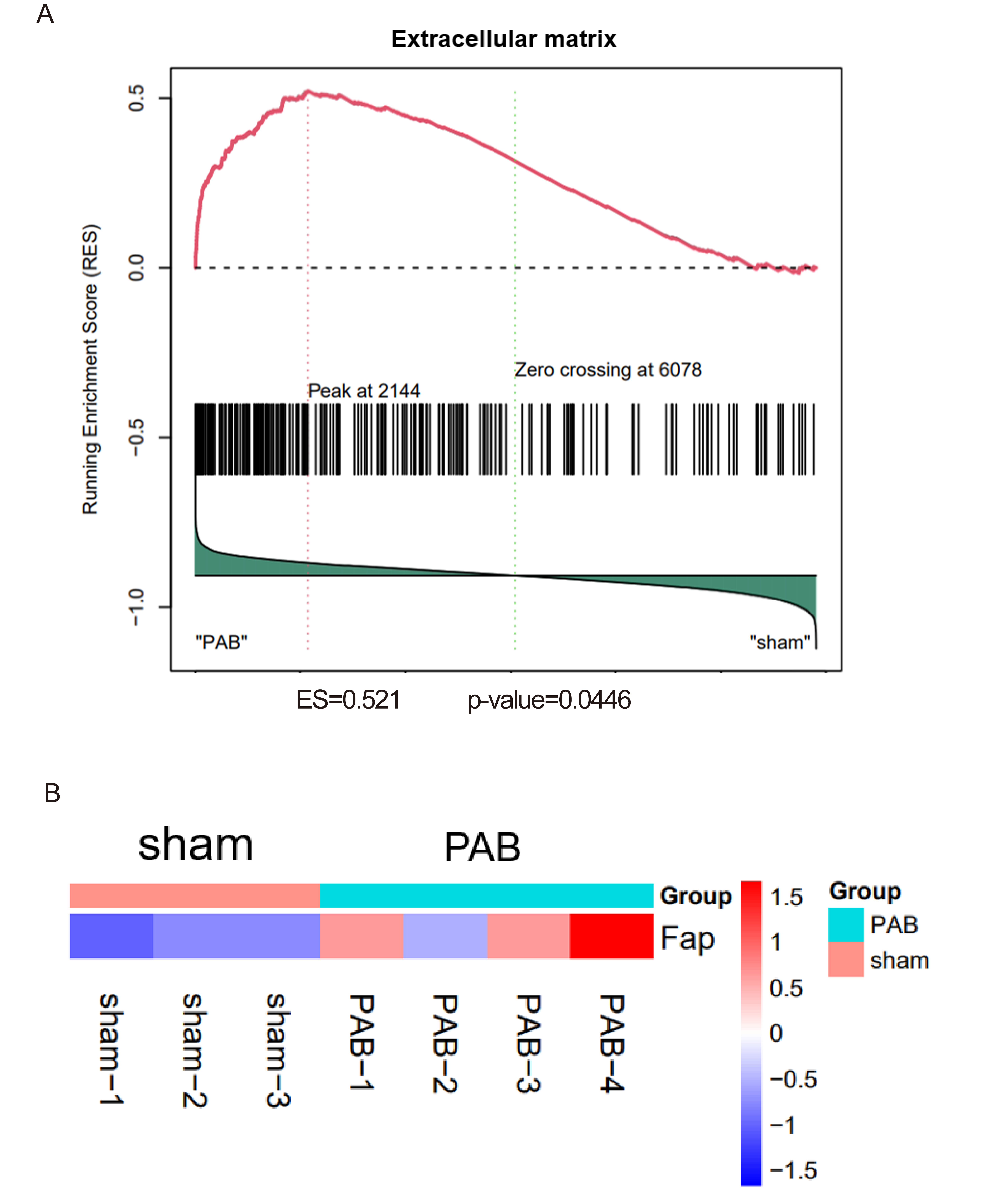

Supplement: Supplementary file 1 — Supplementary Material 1 [file 12931_2023_2565_MOESM1_ESM.png]
