# Supplementary material for: [18 F] -FAPI-42 PET/CT assessment of Progressive right ventricle fibrosis under pressure overload
Source: Respir Res. 2023 Nov 6;24:270. doi: 10.1186/s12931-023-02565-5 (PMC10626814; doi:10.1186/s12931-023-02565-5)
Supplement: Supplementary file 4 — Supplementary Material 4 [file 12931_2023_2565_MOESM4_ESM.docx]

*Respiratory Research*

**[^18^F] -FAPI-42 PET/CT Assessment of Progressive Right Ventricle Fibrosis under Pressure Overload**

Xiaohui Zeng^1#^, Ruiyue Zhao^2#^, Zhixiong Wu^1#^, Zhuoji Ma^1#^, Chunxian Cen^1^, Shanshan Gao^1^, Wanxian Hong^1^, Yanrong Yao^3^, Kexin Wen^3^, Shangwei Ding^4^, Jian Wang^1^, Wenju Lu^1^, Xinlu Wang^2^*****, Tao Wang^1^*****.

^1^State Key Laboratory of Respiratory Diseases, Guangdong Key Laboratory of Vascular Diseases, National Clinical Research Center for Respiratory Diseases, Guangzhou Institute of Respiratory Health, the First Affiliated Hospital of Guangzhou Medical University, Guangzhou, Guangdong, China.

^2^Department of Nuclear Medicine, the First Affiliated Hospital of Guangzhou Medical University, Guangzhou, Guangdong, China.

^3^Guangzhou Medical University, Guangzhou, Guangdong, China.

^4^Department of Ultrasound, the First Affiliated Hospital of Guangzhou Medical University, Guangzhou, Guangdong, China.

^#^These authors contributed equally to this work.

***Corresponding to**:

Dr. Tao Wang (Email: taowang@gzhmu.edu.cn), State Key Laboratory of Respiratory Diseases, Guangdong Key Laboratory of Vascular Diseases, Guangzhou Institute of Respiratory Health, the First Affiliated Hospital of Guangzhou Medical University, Guangzhou, Guangdong, China. Tel: 86-020-83205036.

Dr. Xinlu Wang (Email: 71lu@163.com), Department of Nuclear Medicine, the First Affiliated Hospital of Guangzhou Medical University, Guangzhou, Guangdong, China.

Supplemental Table 1. The primer sequences used in qPCR.

|  | Forward (5′-3′) | Reverse (5′-3′) |
| --- | --- | --- |
| 18s | TATGGTTCCTTTGGTCGCTCGCTC | TCTGATAAATGCACGCATCCCCCC |
| ANP | ATGGGCTCCTTCTCCATCAC | TTTCTCCTCCAAGGTGGTC |
| BNP | AGTCTCCAGAACAATCCACGATGC | CCGGAAGGCGCTGTCTTGAG |
| Col1a1 | CAGAGCACCATTTTCCAAAGCA | GGTACAGAGTCTCTTGCTTCCT |
| Col3a1 | ATATCAAACACGCAAGGCCA | TTGCTGGGGTTTCAGAGAGTT |
| CD31 | CTCCTAAGAGCAAAGAGCAACTTC | TACACTGGTATTCCATGTCTCTGG |
| VE-cadherin | ACCAGTGACAGAGGCCAATACT | GGCCTCCACAGTCAGGTTATAC |
| Fibronectin | TCAGCTGTACCATTGCAAATC | TGGTGTCCTGATCATTGCAT |
| Vimentin | TCCCTGAACCTGAGAGAAAC | ATCGTGGTGCTGAGAAGTC |
